# International Medical Congress—Fiction Versus Facts

**Published:** 1886-01

**Authors:** 


### THE INTERNATIONAL MEDICAL CONGRESS—FICTION VERSUS FACTS.

*Editors Atlanta Medical and Surgical Journal:*

The *Medical News*, of Philadelphia, of December 12, 1885, under the heading of "The Future Congress," says: "It is generally conceded that the miscarriage of the plans for the organization of the proposed International Medical Congress, at Washington, has inflicted an injury upon the profession which is wide spread."

So far from this being as stated "generally conceded," or there being a "miscarriage of plans," there are now hundreds in the profession in the United States (Philadelphia being a very small portion of this vast country) so confident of the success of the Congress, as to have already accepted their duties, and are at this moment actively engaged in preparing work for its sections.

"The time which should have been spent in active preparation is now half gone, and there is no actual preparation for the meeting."

On the contrary, the "General Committee on Organization" have met several times, traveling hundreds of miles to different meetings; have selected the general officers and presidents of sections; arranged seventeen sections, and in accordance with instructions turned the further details over to an able Executive Committee of seventeen prominent men. This "Executive Committee" had two full meetings on September 23 and November 18, 1885, in New York and settled the "Preliminary Organization," and printed circulars giving the rules, etc., of the Congress in parallel columns in English, French and German languages. These circulars are being widely disseminated in Europe and the United States.

The *News* goes on to say: "It is useless to consider the causes which have produced this existing lamentable condition of affairs."

This is true; but it cannot be forgotten that the main cause has been the vicious opposition of the "Philadelphia *Medical News*" and the "New York *Medical Record*," and "New York *Medical*

*Journal*," that have continuously misstated facts, endeavoring to impair the action of the profession through the American Medical Association, and striving to create a favorable sentiment towards the formation of a *new* "National Medical Association."

The "Philadelphia *Medical News*" also suggests, in its great anxiety for the success of the Congress, that "a solution of the difficulty, that a profession in Philadelphia" (as it understands it) "have formally pointed out, is to be found in the union of the present "Executive Committee" with the "Original Enlarged General Committee." Yet when the "Executive Committee" lately gave official notice that they had increased their number by electing six of the more prominent members of the opposition, the result was that those elected declined to accept the appointments unless the Executive Committee would "unite with the Original Committee and re-commence the organization *de novo*." Truly, "those whom the gods wish to destroy they first make mad." These physicians are willing to sacrifice their professional honor by failing to support an Executive Committee of unexceptionable men, who are striving to make good the invitation, which the editor of the Philadelphia *Medical News*, and others of the Original Committee, carried to Copenhagen, in the name of the profession of the United States, asking them to hold their next Congress at Washington, and "*assuring them of a hearty welcome*." Perhaps before the Congress is over these dissatisfied doctors will recognize the fact that there *is* medical talent elsewhere than in the limited district known as the Eastern States. The vast South and West will be heard from in a way that will show how great was the error created by the course taken by the original committee of eight, and a large body of their profession will charge them with disloyalty to the great interests entrusted to their judgment and their honor in "assuring a a hearty welcome" to our European brethren.

Respectfully,

CORRESPONDENT.

December 18, 1885.

